# Commentary: Special Issue: Current Understanding of Colorectal and Pancreatic Cancers

**DOI:** 10.1093/mutage/geae009

**Published:** 2025-03-16

**Authors:** Pavel Vodicka, Ludmila Vodickova

**Affiliations:** Department of Molecular Biology of Cancer, Institute of Experimental Medicine of the Czech Academy of Sciences, Videnska 1083, 142 00 Prague, Czech Republic; Institute of Biology and Medical Genetics, 1st Faculty of Medicine Charles University, Albertov 4, 128 00 Prague, Czech Republic; Biomedical Centre, Faculty of Medicine in Pilsen Charles University, Alej Svobody 76, 323 00 Pilsen, Czech Republic; Department of Molecular Biology of Cancer, Institute of Experimental Medicine of the Czech Academy of Sciences, Videnska 1083, 142 00 Prague, Czech Republic; Institute of Biology and Medical Genetics, 1st Faculty of Medicine Charles University, Albertov 4, 128 00 Prague, Czech Republic; Biomedical Centre, Faculty of Medicine in Pilsen Charles University, Alej Svobody 76, 323 00 Pilsen, Czech Republic

More than a decade has passed since the last Special Issue in *Mutagenesis* (Vol. 27, No. 2, 2012) was published, inspired by the COGENT (Colorectal Cancer Genetics) consortium and dedicated to exploring individual susceptibility to colorectal cancer (CRC). CRC, as well as pancreatic ductal adenocarcinoma (PDAC), represents severe malignancies, causing tumor-related deaths worldwide (CRC was diagnosed in 1.8 million individuals, causing 900 000 deaths; PDAC was diagnosed in 495 773 individuals and responsible for 466 003 deaths in 2020 worldwide [1–3]).

Over the last decade, several important genome-wide association studies (GWASs) have emerged, addressing the individual susceptibility to the disease in general [4,5] as well as other serious aspects, such as differences in genetic architecture regarding the anatomic localization of the tumor [6] and an early onset of CRC [7]. The involvement of microbiome and fecal microRNA (miRNA) signatures in distinguishing precancerous adenomas from CRC has recently been published as well [3]. PDAC has an incidence that almost matches its mortality, but only a small number of risk factors and 33 susceptibility loci have been identified so far. The relative scarcity of PDAC hampers research on the genetic mechanisms contributing to the disease and precludes small monocentric studies from being successful. The PANcreatic Disease ReseArch (PANDoRA) consortium, bringing together 29 basic and clinical research groups, has contributed to the discovery of 25 susceptibility loci, a feat that will be instrumental in stratifying the population by risk and optimizing preventive strategies [2].

While the risk factors for PDAC remain enigmatic, Murphy *et al.* recently summarized convincing evidence on the role of inflammation, lipid peroxidation, oxidative stress, and metabolic dysfunction in the onset of CRC and its development [8].

In this Special Issue of *Mutagenesis*, we have collected five reviews and four experimental articles on CRC and PDAC. The review articles provide an overview on several aspects from DNA repair and genomic instability, nonspecific chromosomal aberrations (CAs), genomic profiling of both cancers, telomere homeostasis, and inflammation and gut barrier function versus CRC risk.

The first review article by Andarawi *et al.* focuses on the essential topics of genome integrity, DNA damage and its repair in proper cellular functions, and maintenance of physiological processes. Unless appropriately repaired, DNA damage can lead along with telomere homeostasis and DNA replication and mitosis [9] to genomic instability, which is associated with the development and progression of various human diseases. The authors surveyed the involvement of DNA damage response gene alterations in human normal physiologic processes and diseases, including cancer, neurodegenerative diseases, and immunological disorders. Interestingly, DNA damage and DNA damage response pathways are involved in several distinct pathologies, stretching from proliferative to degenerative diseases; however, the switch and mechanisms of particular etiopathogenetic processes are not sufficiently elucidated [10].

Eid *et al.* evaluated the germline findings by genomic profiling in two frequent gastrointestinal malignancies, PDAC and CRC, posing severe health problems and exhibiting high incidence and mortality. The review discusses various aspects affecting prognosis (as overall survival, event-free survival, and therapy prediction) in both PDAC and CRC. The universal multigene panel testing (83 genes) was shown to detect pathogenic germline variants (PGVs) in 12.5% of patients (regardless of cancer type, sex, family history of cancer, age at diagnosis, stage of disease, etc.). This advocates for the methods that increase the detection of PGVs. The authors suggest the approach of evaluating the variant allele frequency, i.e. the number of variant reads divided by the number of total reads, serving as an indicator for germline testing outside the current indication criteria. The article reports the evaluation of patients with advanced and metastatic tumors who underwent somatic panel testing at the University Hospital Brno, Czech Republic. In patients with PDAC and CRC, the authors describe their clinical characteristics and prevalence of gene variants suspected to be PGVs (Cohort A). In addition to this, results from the local genetic database of patients diagnosed with PDAC and CRC who underwent germline sequencing according to standard screening criteria (Cohort B) are presented. The authors discuss comprehensively obtained gene variants in relation to prognostic and predictive markers in sporadic PDAC and CRC neoplasia [11].

The review article of Hemminki *et al.* highlights that monitoring of CAs is so far the only method of assessing cancer risk in healthy human populations. Previous studies have shown the association of the frequency of CAs in peripheral blood lymphocytes and cancer risk. The mechanisms for CA formation are likely due to unrepaired or insufficiently repaired DNA double-strand breaks or other DNA damage, and additionally telomere shortening. The authors discuss the types of CAs assayed for by the conventional techniques and aspects of interpreting the results. When germline genetic variation was determined in a GWAS in healthy individuals in relation to occupational and smoking-related exposure compared with nonexposed referents, several candidate genes from DNA damage response/repair pathways, apoptosis, cell proliferation, angiogenesis, and tumorigenesis, and autistic traits were disclosed. Dedicated studies on 153 DNA repair genes revealed associations for about 30 genes, expression of which could be modulated by the implicated variants [12]. Indeed, the topic of nonspecific CAs in cancer risk and onset is far from being explored and the entire field may be more attractive by employing automated performance steps and incorporating artificial intelligence scoring methods.

The review of Campa *et al.* analyses telomere length measurement in leukocyte (LTL) in relation to PDAC risk. Despite the results are not entirely convincing, the short LTL is often associated with an increased PDAC risk. GWAS revealed several single nucleotide polymorphisms (SNPs) in the telomerase reverse transcriptase gene as a susceptibility loci for PDAC. The authors provide an overview of the most relevant associations between SNPs in telomere-associated genes and PDAC, to address the question of whether shorter or longer LTL is associated with PDAC risk and which one has a prognostic value [13].

In the study of Tomasova *et al.*, LTL was monitored in 198 patients with CRC over time since the diagnosis in 6-month intervals for 18 months. The authors observed a pronounced LTL decrease 12 months after the diagnosis; this decrease followed different trajectories according to tumor location. The LTL dynamics, however, suggested the likely different roles of various white blood cell forms [14]. White blood cells may play a role in tumor immunology and further studies involving LTL measurement in sorted white blood cells are warranted.

The review by Mandle *et al.* provides an overview of the first comprehensive pathway-based evaluation of the functional and tagging SNPs within inflammation and gut barrier-related pathways and genes, alone, and in combination with circulating biomarkers and CRC risk. The authors conducted exploratory analyses to investigate whether these associations differ by tumor site and sex. Novel findings include potential associations between genetic variation in genes related to lipopolysaccharide (LPS) response and tolerance, and CRC risk [15]. The field of microbiome and microenvironment in gastrointestinal cancers is rapidly expanding. Evidence suggests that these factors are essential for cancer onset, diagnostics, and its prognosis and prediction [3].

The experimental article by Hackert *et al.* addressed (i) a pleiotropy scan for discovering new susceptibility loci for PDAC, (ii) fecal miRNA profiles in mucinous CRC, (iii) miRNAs 122-5p and 142-5p and their role in chemoresistance of CRC patients, and (iv) homologous recombination repair in relation to carboplatin response in ovary cancer, treated like CRC with platinum derivatives. The authors report pleiotropic variants (i.e. genetic polymorphisms influencing more than one phenotype) in their association with cancer risk. On the basis of the hypothesis that variants already associated with a least one trait have a higher probability of association with other traits, the variants were tested in the two-phase study (16 055 PDAC cases and 212 149 controls). The meta-analysis of the two phases showed two loci (10q21.1-rs4948550 (*P* = 6.52 × 10^−5^) and 7q36.3-rs288762 (*P* = 3.03 × 10^−5^) potentially associated with PDAC risk. Although correction for multiple testing precluded formal statistically significant association of the SNPs, the two SNPs showing the best associations with PDAC risk (having also pleiotropic nature and association with various human traits including CRC) merit further investigation through fine mapping and ad hoc functional studies [16]. This applies especially in the context of relatively poor genetic mapping of PDAC.

The article by Naccarati *et al.* postulates that diagnostic performance based on surrogate tissues like stool may be affected by CRC heterogeneity. The mucinous histotype represents CRC with a peculiar molecular program and unfavorable disease progression. The authors investigated whether miRNA profiles in the stool of patients with CRC differ with patients with mucinous histopathological cancer compared with non-mucinous cancer. The authors found 49 differentially expressed (DE) stool miRNAs in CRC with mucinous morphology and 61 in non-mucinous CRC. Among the 48 stool DE miRNAs observed in patients with mucinous CRC, 20 were also DE in comparison between tumor and adjacent mucosa tissue. This study highlights miRNAs specifically altered in CRC with mucinous morphology; however, the performance of stool miRNA signature in the accurate distinguishing CRC cases from controls is relatively low for the mucinous histological subtype [17]. The questions related to the diagnosis, cancer risk, prognosis, and prediction, involving microbiota and the microenvironment, require further targeted investigations.

The last two experimental articles are dedicated to the prediction of therapeutic response in patients with cancer. The former dissects the effect of miRNAs in acquired chemoresistance. The authors focus on miRNA-122-5p and miRNA-142-5p and their involvement in the progression of colon cancer and modulating effects in 5-fluorouracil (5-FU) chemoresistance development. Marked differences in the expression levels of both miRNAs between individual samplings over time were observed. Patients with colon cancer without relapse exhibited higher expression of miRNA-122-5p at follow-up intervals. Out of miRNA-122-5p-validated targets, 25 targets (such as GLUL, GPR161, and TRIM29) were significantly differentially expressed between nonresistant and 5-FU-resistant cell lines [18]. Potential therapeutic targets of miRNA-122-5p related to 5-FU-based chemoresistance were identified by analysis of 5-FU-resistant cell lines, but focused mechanistic studies are still needed in the concept of personalized medicine. Interestingly, miRNA-122-5p could be used as a predictive biomarker of tumor relapse in patients with colon cancer.

In the former study of Horak *et al.*, it was postulated that miRNA-140 targets MRE11 and modulates CRC prognosis. The lower expression of miRNA-140 was associated with the metastatic phenotype and poor progression-free survival. Both *in vitro* and *in vivo* results suggest that miRNA-140 may act as a tumor suppressor and plays an important role in double-strand break repair and, consequently, CRC therapy response, particularly toward platinum derivatives [19]. The further experimental study of Horak *et al.* investigates whether it may be possible to utilize MRE11 (a constituent part of homologous recombination repair and responsible for the repair of carboplatin (CbPt)-induced DNA damage, particularly DNA crosslinks) in overcoming CbPt chemoresistance in ovarian cancer. Lower expression of MRE11 was associated with better overall survival in a cohort of patients with ovarian cancer treated with platinum drugs (TCGA dataset, *P* < .05). *In vitro* analyses revealed that high expression of homologous recombination repair genes drives the CbPt chemoresistance in our CbPt-resistant cell line model. In this context, the inhibition of homologous recombination repair by Mirin not only significantly increased sensitivity to carboplatin but also rescued the sensitivity in the CbPt-resistant mode. These data evince that Mirin inhibition of MRE11 may represent a way to overcome ovarian cancer resistance. Additional experiments are, however, required prior to the application of this concept in personalized cancer therapy [20]

In conclusion, the Special Issue *Current Understanding of Colorectal and Pancreatic Cancers* addresses several important points in the risk, onset, progression, prognosis, and prediction of two important malignancies, CRC and PDAC. While both the reviews and experimental articles addressed risk factors, cancer genetics and, to some extent, tumor microenvironment and microbiome, some large areas need to be explored. First, we have to note that CRC in contrast to PDAC is understood in greater depth, but the field requires deeper insight into intra-tumor heterogeneity, adenoma to carcinoma transition, and cancer progression into metastatic processes, and immune system involvement in the onset of CRC and progression. All these aspects are critical for the identification of improved therapeutic targets and regimens. Regarding PDAC, the situation is less optimistic. The risk factors are not yet defined, and diagnosis often occurs at a late stage, when cancer is in an advanced, untreatable, stage. However, it is encouraging that progress has been made in understanding PDAC genetics and several laboratories are working on biomarkers for early diagnosis.
